# Barriers, facilitators, and potential strategies for increasing HPV vaccination: A statewide assessment to inform action

**DOI:** 10.1016/j.pvr.2017.11.003

**Published:** 2017-12-07

**Authors:** Kathleen B. Cartmell, Jennifer Young-Pierce, Shannon McGue, Anthony J. Alberg, John S. Luque, Maria Zubizarreta, Heather M. Brandt

**Affiliations:** a Medical University of South Carolina, Hollings Cancer Center, Charleston, SC, United States; b Medical University of South Carolina, College of Nursing, Charleston, SC, United States; c Medical University of South Carolina, Department of Gynecologic Oncology, Charleston, SC, United States; d Medical University of South Carolina, College of Medicine, Charleston, SC, United States; e Medical University of South Carolina, Department of Public Health Sciences, United States; f University of South Carolina, Arnold School of Public Health, Columbia, SC, United States

**Keywords:** HPV, HPV vaccines, Cervical cancer, Prevention, Health systems, Barriers, Facilitators, Strategies, South Carolina

## Abstract

**Objective:**

The objective was to investigate how state level strategies in South Carolina could maximize HPV vaccine uptake.

**Design:**

An environmental scan identified barriers, facilitators, and strategies for improving HPV vaccination in South Carolina. Interviews were conducted with state leaders from relevant organizations such as public health agencies, medical associations, K-12 schools, universities, insurers, and cancer advocacy organizations. A thematic content analysis design was used. Digital interview files were transcribed, a data dictionary was created and data were coded using the data dictionary.

**Results:**

Thirty four interviews were conducted with state leaders. Barriers to HPV vaccination included lack of HPV awareness, lack of provider recommendation, HPV vaccine concerns, lack of access and practice-level barriers. Facilitators included momentum for improving HPV vaccination, school-entry Tdap requirement, pharmacy-based HPV vaccination, state immunization registry, HEDIS measures and HPV vaccine funding. Strategies for improving HPV vaccination fell into three categories: 1) addressing lack of awareness about the importance of HPV vaccination among the public and providers; 2) advocating for policy changes around HPV vaccine coverage, vaccine education, and pharmacy-based vaccination; and 3) coordination of efforts.

**Discussion:**

A statewide environmental scan generated a blueprint for action to be used to improve HPV vaccination in the state.

## 1 Introduction

HPV vaccination is a major cancer prevention breakthrough, but the full public health benefits of these vaccines have yet to be realized in the US. The Advisory Committee on Immunization Practices (ACIP) recommends vaccination at ages 11–12, but only 63% of US females ages 13–17 have received the first HPV vaccine dose, and only 42% have received all three doses needed for full protection [1]. Among boys ages 13–17, first and third dose vaccination is only 50% and 28% respectively [1]. HPV vaccination rates are even lower in South Carolina (SC) than they are nationally, with 1st and 3rd dose coverage respectively at 54% and 34% among girls and 29% and 16% among boys [1]. Compared to other states in the US, SC ranks 39th and 41st respectively for 3rd dose HPV vaccine coverage for girls and boys [1].

Barriers to HPV vaccination at the national level include factors related to vaccination in general and those specific to HPV vaccine. These occur at the level of the patient, provider, and health system or policy environment. At the patient level, barriers include: lack of recommendation by the provider [2], [3], [4], lack of knowledge about the vaccine and HPV-related diseases [5], [6], [7], concerns about vaccinating an adolescent against a sexually transmitted infection [2], [8], [9], lack of conviction that the vaccine is essential (especially for males) [10], [11], [12], and concerns about vaccine safety and costs [2], [9], [10], [13], [14]. At the provider level, barriers include: lack of understanding about HPV-related diseases (especially for males) [15], [16], safety concerns [17], [18], concerns about vaccine reimbursement [18], [19], personal attitudes [17], discomfort talking to parents and children about a topic related to sexual behavior [20], concerns about parental resistance [17], [18], [21], preference for vaccinating older vs. younger adolescents [22], [23], lack of vaccine reminder and recall systems [24], [25], and lack of time to provide education about the vaccine [18]. Barriers at the health policy level include a lack of coverage of the vaccine among some populations [26], [27], [28], [29], [30], [31] and a lack of legislation to make the vaccine mandatory for school entry in most US states [32].

In response to the low national uptake of HPV vaccination, the 2014 President's Cancer Panel Report identified underuse of HPV vaccines as a “serious but correctable threat to progress against cancer [33].” The report made three key recommendations to overcome barriers to HPV vaccination across the patient, provider, and health system/policy levels: 1) use of multi-level intervention strategies; 2) thoughtful collaboration and coordination of resources and services among diverse stakeholders; and 3) state-specific strategies that account for factors such as the preferences and needs of state residents and existing health systems, resources and policies.

After publication of the 2014 report, the Hollings Cancer Center in South Carolina (SC) was one of 18 sites funded by the National Cancer Institute (NCI) to carry out environmental scans to evaluate the barriers, facilitators, and potential strategies needed to improve state-level HPV vaccination rates [34]. HPV vaccination rates are lower in SC than they are nationally. In SC, 1st and 3rd dose coverage is 54% and 34% among girls and 35% and 21% among boys [1]. The purpose of this evaluation was to identify contextually-appropriate intervention strategies that may be feasible, acceptable, and effective for improving HPV vaccination in SC.

## 2 Methods

### 2.1 Design

This was an NCI-funded project to identify contextually appropriate strategies for improving HPV vaccination uptake in SC by conducting an environmental scan of the barriers, facilitators, and strategies needed for improving HPV vaccination. Thirty-four key informant interviews were conducted with state leaders who represented diverse organizations that have potential to impact HPV vaccination policies and practices. On average, interviews lasted 40 min (range: 30–60 min). The interviews took place during January through June of 2015. Our two interviewers were a PhD-trained cancer control researcher and a gynecological oncologist; each had expertise in cancer control, HPV vaccination and qualitative interviewing. After the interview, participants completed a self-administered demographic survey. Participants were not compensated. A thematic content analysis approach was utilized [35]. The Medical University of South Carolina Institutional Review Board reviewed the study protocol and deemed this project as program evaluation, obviating the need for IRB approval.

### 2.2 Guiding framework

The Socio-Ecological Model (SEM) was used as the conceptual model for characterizing the multiple levels of influence on HPV vaccination. The SEM was developed based on the understanding that most public health problems are too complex to be conceptualized and addressed from any single level analyses [36]. The SEM describes five nested levels of influence: individual (knowledge, attitudes, skills), interpersonal (social network), organizational (environment, ethos), community (cultural values, norms), and public policy/environmental context. This model informed the development of our interview guide that focused on characterizing barriers and facilitators, partnerships and promising intervention strategies across each level of the SEM to address underutilization of the HPV vaccine.

### 2.3 Participants and setting

Purposive sampling was used to recruit leaders from organizations across the state who had the potential to influence statewide HPV vaccination policies and practices. To select stakeholders, the research team held a brainstorming session to create a list of key organizations in the state whose missions could help to improve HPV vaccination practice and policy; and from this list, the name and contact information for each organization's leader was identified. Participants were recruited from this list via a series of three email invitations, followed by two personal phone calls to those who had not responded to email invitation. Each of these contact attempts were spaced approximately two weeks apart.

Table 1 provides a summary of the leaders from the state organizations who participated in the interviews. These organizations included public health (immunization and cancer control programs), provider organizations (e.g. medicine, family practice, pediatrics, federal health centers, pharmacy, school nurses), large insurers (Blue Cross Blue Shield, Medicaid), state quality improvement collaboratives (SC American Academy of Pediatrics QI collaborative, SC medical home QI collaborative), K-12 schools, three large state universities (Clemson, University of South Carolina, South Carolina State), non-profit and grassroots organizations that target cancer prevention (American Cancer Society, SC Cancer Alliance, Cervical Cancer Free SC and the Witness Project cervical cancer prevention project) and adolescent health (Planned Parenthood, SC Campaign to Prevent Teen Pregnancy), and state legislators. All organizations invited to participate in interviews completed interviews, with the exception of two legislators who were known HPV vaccination opponents and one state university, who did not respond to our invitations. Most interviews were conducted in person, with three interviews conducted by phone, based upon stakeholder preference.

**Table 1:** Characteristics of key informant interview participants (n = 34).

**Characteristic**	**Category**	**Number (%)**
**Age Group**	Under age 45	13 (38.2%)
	45–54	9 (26.5%)
	55–64	9 (26.5%)
	65 or older	3 (8.8%)
**Race**	Non-Hispanic Black	6 (17.6%)
	Non-Hispanic White	28 (82.4%)
**Gender**	Male	9 (26.5%)
	Female	25 (73.5%)
**Education Level**	Some College Education	2 (5.9%)
	College Degree	3 (8.8%)
	Graduate Degree or Above	29 (85.3%)
**Stakeholder Role**	State Health Department	5 (14.7%)
	State Physician Organizations	5 (14.7%)
	State Pharmacy Organization	1 (2.9%)
	State Insurers	3 (8.8%)
	State Quality Improvement Collaboratives	3 (8.8%)
	State Department of Education	1 (2.9%)
	School Nurses Association	1 (2.9%)
	University School Health Programs	4 (11.8%)
	State Legislators	3 (8.8%)
	Grassroots Cancer Prevention Organizations	6 (17.6%)
	Grassroots Adolescent Health Organizations	2 (5.9%)
**Service Area**	Statewide	34 (100%)

### 2.4 Data collection, management and analysis

Key informant interviews were the primary data source. The semi-structured interview guide, as shown in Appendix A, was developed by investigators with expertise in evaluation, qualitative research, HPV, and cervical cancer, and was pilot tested with two cancer control professionals prior to use. The interview guide focused on identifying best practices for improving HPV vaccination in SC. Questions pertinent to areas of expertise of all participants queried: a) stakeholder perceptions about HPV vaccination; b) barriers and facilitators to HPV vaccination; c) recommended strategies for improving vaccination rates; and f) key partnerships and discussions needed for developing strategies to maximize HPV vaccination rates. Optional interview modules were developed for use by stakeholder type on the following topics: clinical reminder and recall systems, clinician HPV vaccination perceptions and practices, HPV vaccination coverage by insurers, public health immunization program, HPV-related legislation, school-based HPV vaccination, and pharmacy-based HPV vaccination.

Interviews were audiotaped and transcribed. Transcripts were checked for accuracy by a second person. The data collected were highly structured based on the specific purpose of the study. Two researchers reviewed a sample of the interview transcripts independently to create a joint codebook to guide formal analysis. The codebook was used to code all transcripts, and emergent themes were added as needed, such as when themes arose from tailored interview questions with specific participants. The preliminary results were circulated to all co-authors and additional researchers for review and discussion. This iterative process strengthened the ability of the research team to optimize valid interpretations of the interviews.

## 3 Results

The characteristics of the 34 individuals who participated in the key informant interviews are shown in Table 1. All participants had work roles in which they served or represented stakeholders at a state level. Several themes emerged from the interviews. The key results for the environmental scan centered on barriers, facilitating factors, and strategies to increase HPV vaccination rates in SC; each is presented in detail below.

### 3.1 Barriers

The main barriers identified were lack of HPV awareness among the general public, lack of provider recommendation, concerns about HPV vaccination, lack of access among young adults and underinsured adolescents, reimbursement barriers for pharmacy administration, difficulty completing the three-dose series, and practice-level barriers (Table 2).

**Table 2:** Barriers to HPV vaccination reported by participants.

**Themes**	**Quotes**
**Lack of HPV awareness among the general public**	
Absence of systematic messaging to promote accurate information about HPV vaccination	– One of the biggest barriers is lack of promotion; No big state push to say vaccinate your kids like they do for flu vaccine. – When I drive home I see a sign for ovarian cancer about going to get yourself checked out. We don’t have anything like that for cervical cancer…nothing to just tell people, hey you can avoid getting cancer. – So much intentional misinformation. Consumers don't know how to differentiate misinformation from scientific information.
Lack of awareness of importance of HPV vaccination among key stakeholders in state	– All the big systems! You have a health department that is not congenial to push the envelope with pushing the vaccine protocols. You have a legislature that is obstinate at best and ludicrous at worst. – We have legislators who don’t support the vaccine. – Stronger support for the HPV vaccine is needed at the state health department.
Lack of awareness among parents/patients	– People, especially in rural areas, don’t understand the link between HPV and cervical cancer. – I suspect there is a lot of misinformation among the parents of these children we are trying to vaccinate. – I teach at a college and did a session on sexually transmitted disease; most of them hadn’t heard of the HPV vaccine.
Parental lack of awareness compounded by poor provider vaccine endorsement	– Frankly I think providers are a barrier; providers not recommending vaccination due to their own personal beliefs. – I inherited patients from a coworker who retired and learned she’d been recommending against the HPV vaccine. I learned from that one negative recommendation from someone patients have known & trusted is nearly irreversible no matter how hard I tried. – My pediatrician did not bring it up. I had to request it.
Lack of awareness males need vaccine	– Boys are not getting the message that the vaccine is for them…The bill is called the Cervical Cancer Prevention Act instead of HPV, but you don’t think of an adolescent boy taking that – it's for cervical cancer.
**Lack of provider recommendation for HPV vaccination**	
Provider lack of awareness of guidelines	– Lack of physician education – Need not only education of patients, but providers. Who needs the vaccine? Why do they need it? How do you assess these needs?
Provider discomfort discussing the topic	– Some providers don't feel comfortable giving clear recommendations for HPV vaccination. They have inadvertently been affected by the anti-vaccine lobby. Instead of saying you need your HPV vaccine today; they shy away. – Some physicians, even in my practice, wait until age 15 or 16 to recommend the vaccine.
Perceptions about time required to present HPV vaccination	– Providers may be hesitant to recommend the vaccine because they have a very limited amount of time with patients and don't want to get caught up in a 15- minute conversation about one vaccine. – Some FQHC providers have said that the main reason they don't ask these questions is because what if the patient says yes, I'm having sex. Now they're into a 45-min discussion and they just don't have the time for that.
Difficult to address preventive care issues because adolescents tend to only visit physicians for acute health issues	– If you have a 16-year-old coming in with a sore throat, you could take 5 min to ask if she's having sex. But that makes them freak out. I's the same with a 9-year-old coming in with a sports injury who's not being asked if they've been vaccinated for HPV. – Pediatricians are often concerned with childhood illnesses rather than a disease that could affect them later in life. – Most adolescents don't come in for well visits. – The vaccine is recommended at around age 12. Not many other vaccines at that time.
**Concerns about HPV vaccination**	
Sexually transmitted nature	– As a global comment in South Carolina, the HPV vaccine is tied to sex. It is not tied to cancer prevention. – We are the Bible Belt, and people are not comfortable with their children being sexually active. – HPV vaccination got a bad rap: you’re encouraging my child to have sex. – The #1 barrier is that we live in a very conservative state with strong family values. It's gotten out this will promote promiscuity.
Sexually transmitted nature significant in context of recommended age for vaccination	– It is one of the more difficult vaccines because of the moral issues that come with an STD and vaccinating prepubescent children. – My child's not going to have sex so why vaccinate them for an STD. This is a barrier to getting younger adolescents vaccinated. – There are parents who want to wait until a child older to get it, but the younger the child is, the better response to the vaccine. – Some physicians, even in my practice, wait until age 15 or 16 to recommend the vaccine.
Uncertainty about safety of vaccines and HPV vaccine in particular	– People are wary of any vaccine and associated risks. This is a big topic in the media now. – I had a talk with colleagues with children the age for the vaccine; they felt they didn’t have enough vaccine info. – I think we see barriers from anti-vaccination groups who say they are concerned that there is not enough data. – From the other side there are questions: How old is the vaccine? Is this a clinical trial? Are we guinea pigs?
Greater concern among African American (AA) parents in regards to new interventions	– There is distrust in the [AA] community about medical treatments of any kinds. – I’ve spoken to a few folks in the AA community and unfortunately there is still culturally a fear of clinical trials. – They started an HPV vaccine program with minority schools. The parents at the schools took that as "You're experimenting on our children. Why are you doing this? Are you saying our children are more sexually active than the white schools?”
**Lack of access**	
Cost barriers	– Yes, the cost is somewhat of a hurdle; let's say it is $150 per shot, that's $450. – Lack of public funding is a problem. A lot of people don’t have money to go to a private doctor for the vaccine. – Does the patient have the ability to go to a provider who can give the vaccine? Do they have insurance to pay it?
HPV vaccination not included in SC Vaccine Program for adolescents	– Our state vaccine program excludes HPV vaccination, so children who are underinsured can receive any other vaccine recommended by the CDC except the HPV vaccine. This affects 2400 children every year. – At least every few weeks I see a child covered by the state vaccine program, which does not cover HPV vaccine. That is a big problem. Physicians are not likely to recommend it to children who are not covered for the vaccine. – DHEC can cover the vaccination without legislation, but want to wait for legislation direction to do this.
Lack of coverage for HPV vaccination among young adults	– Medicaid doesn't cover the HPV vaccine for children after age 18. They are within the recommended vaccine age range, but Medicaid won't cover it. So people can’t access the vaccine when they are still eligible to receive it. – Third-party reimbursement, we have to improve that. Our state health plan on campus won't cover HPV vaccine.
**Practice-level barriers**	
Lack of suitable recall systems for follow-up doses in practices	– Figuring out how to do population management is tricky; most IT systems do not have population management systems in them. – For some centers, staff time is the limitation to create reminder and recall templates – Some who participated in QI projects use recall systems now, but most don't. That is why 3rd dose rates are low. – It is technologically difficult to get a child the first dose and then get them back in for the second and third doses.
Cost of administering HPV vaccine	– Some vaccines you get reimbursed less than the actual vaccine cost. Larger practices have more power to negotiate these costs. – There has definitely been an issue with the cost of maintaining and storing all vaccines in pediatrician offices. – Administrative fees have come up. Some clinics have to carve out vaccines because they don't get full cost back.
Pharmacy reimbursement	– An issue in the pharmacy community is that not all insurance companies will reimburse pharmacists.

#### 3.1.1 Lack of HPV awareness among the general public

Participants reported a wide range of barriers related to HPV vaccine awareness among the general public. A commonly reported barrier was that there has been a total absence of systematic messaging to promote accurate information about HPV vaccination. Stakeholders made comments such as “One of the biggest barriers is lack of promotion; No big state push to say vaccinate your kids like they do for flu vaccine” and “When I drive home I see a sign for ovarian cancer about going to get yourself checked out. We don’t have anything like that for cervical cancer…nothing to tell people, hey you can avoid getting cancer.”

Attempts in the state to increase awareness of HPV vaccination among the general public and parents, such as the 2016 Cervical Cancer Prevention Act, had not been supported widely, according to participants. The Cervical Cancer Prevention Act, designed to educate middle school parents about HPV vaccination and extend coverage for the vaccine, was stalled in the legislature at the time of the interviews. Concerns about lack of awareness even extended to key stakeholders, such as legislators and public health officials in the state, those with influence to modify policies and practices for addressing barriers to increase HPV vaccination. One participant explained “We have legislators that don’t support the vaccine.” Another participant described “Stronger support for the HPV vaccine is needed at the state health department.” Another commonly cited barrier was lack of HPV awareness among the general public. Parents were identified as a particular group whose general lack of awareness persisted and required intervention. As described by one participant “I teach at a college and I did a session on sexually transmitted disease; most of them hadn’t heard of the HPV vaccine.” Another participant described “There is a lot of misinformation among the parents of these children we are trying to vaccinate.” Parental lack of awareness was compounded by poor endorsement of the vaccine by health care providers. One participant explained “Frankly I think providers are a barrier. Providers not recommending vaccination due to their own personal beliefs.” Participants with children reported being left to make the decision to vaccinate by themselves and being told that sons did not need the HPV vaccine. As one participant described “My pediatrician did not bring it up. I had to request it.” Several participants suggested that confusion about the need for vaccinating males stemmed from the focus on cervical cancer in the initial awareness campaigns. One participant noted “boys are not getting the message that the vaccine is for them. The bill (in SC) is called the Cervical Cancer Prevention Act instead of HPV. You don’t think of a boy taking that—it's for cervical cancer.”

#### 3.1.2 Lack of provider recommendation

Participants perceived that factors contributing to the deficiencies in provider communication about HPV vaccine were 1) lack of awareness of HPV vaccine guidelines among some pediatricians and family practitioners, 2) providers’ limited comfort in discussing the topic, 3) perceptions about the time required to present HPV vaccination to parents and address their questions, and 4) the tendency of adolescents to visit physicians only for acute health issues making it difficult to address preventive care issues. Consistently, barriers focused on providers either not recommending HPV vaccination or providing inconsistent or ineffective recommendations. As one participant described “If you have a 16-year-old coming in with a sore throat, you could take 5 min to ask if she's having sex. But that makes them freak out. It's the same with a 9-year-old coming in with a sports injury who's not being asked if they've been vaccinated for HPV.” Another participant explained “Pediatricians are often concerned with childhood illnesses rather than a disease that could affect them later in life.” Participants felt these deficiencies in provider recommendation led to missed opportunities to increase HPV vaccination awareness among parents.

#### 3.1.3 Concerns about HPV vaccination among the public

Participants widely reported parental concerns about HPV vaccination, specifically the sexually transmitted nature of HPV infection and uncertainty about safety, as barriers. Participants perceived the sexually transmitted nature of HPV as a greater barrier in the context of the conservative and religious values in SC. As one participant explained “We are in the Bible Belt, and people are not comfortable with their children being sexually active.” The sexually transmitted nature of HPV was identified by participants as a concern that permeated every level: the general public, providers, and policy makers. One participant described the parental perspective as “My child's not going to have sex so why vaccinate them for an STD.” Another participant explained “Some providers don’t feel comfortable giving clear recommendations for HPV vaccination. They have inadvertently been affected by the anti-vaccine lobby. Instead of saying you need your HPV vaccine today, they shy away.” From the policy perspective, another participant noted “We have legislators who don’t support the vaccine.” According to participants, some parents believed that giving the vaccine was tantamount to endorsing sexual activity and in particular were reluctant to vaccinate their children at the recommended age of 11–12 years old. One participant explained her thoughts about how parents perceive the vaccine, stating “HPV vaccination got a bad rap: you’re encouraging my child to have sex.” Participants who were clinicians described how anticipating such concerns caused them to avoid a strong endorsement of the vaccine or delay recommending the vaccine until patients were older. As one participant described “Some physicians, even in my practice, wait until age 15 or 16 to recommend the vaccine.” Concerns around vaccinating for a sexually transmitted virus were perceived by participants to be connected to parental resistance to interventions such as school-based vaccination.

Related barriers included parental concerns about safety of vaccines in general and the perception that the HPV vaccine was new and untested. While HPV vaccines have been available for over ten years, some participants reported that uncertainty about the safety of the HPV vaccine is still an issue. One participant described “I had a talk with colleagues with children the age for the vaccine; they felt they didn’t have enough vaccine info.” Another participant explained “I think we see barriers from anti-vaccination groups who say they are concerned that there is not enough data.” A few participants felt that the African American community held a greater distrust of new vaccines and medical interventions. As one participant described “There is distrust in the AA community about medical treatments of any kinds.”

#### 3.1.4 Lack of access

Lack of access, related to cost, was identified as a barrier to HPV vaccination among underinsured adolescents and young adults due to SC public programs and private insurers not covering the vaccine. Most children in the state had access to HPV vaccination through private insurance, Medicaid, or the federal Vaccines for Children (VFC) program. At the time of the stakeholder interviews, underinsured adolescents could obtain all ACIP recommended vaccines except HPV vaccine through the SC Vaccine Program. This is a program administered by the state health department that covers vaccinations for underinsured children who are ineligible for Medicaid, but who do not have health insurance. As one participant explained “Our state vaccine program excludes HPV vaccination, so children who are underinsured can receive any other vaccine recommended by the CDC except the HPV vaccine. This affects 2400 children every year.” Participants advised that the state health department should add HPV vaccination to the state program to extend coverage to underinsured children. As described by several participants, the health department could add the HPV vaccine within the state vaccine program based on public health authority, but has instead sought “legislative direction” several times to add HPV vaccination to the state program, which had been repeatedly denied by the legislature. Young adults in the catch-up population for HPV vaccination also faced barriers to access, mainly related to insurance coverage. At the time of the interviews, the largest insurer in the state did not cover HPV vaccination for young adults aged 18–26, though other private insurers did. As one participant explained: “Third-party reimbursement, we have to improve that. Our state health plan on campus won't cover HPV vaccine.” Participants representing university health centers indicated that HPV vaccination status was optional on matriculation immunization forms. HPV vaccination in universities is complicated by limitations in access, but these settings have been resourceful in seeking support from pharmaceutical company programs and utilizing referrals to provider settings where there may be more financial support for vaccination.

#### 3.1.5 Practice level barriers

Several participants representing multiple sectors noted the importance of practice-level barriers in low HPV vaccination uptake. A commonly cited barrier was simply that adolescents did not visit the doctor unless they were sick or were in need of sports physicals. One participant stated “Most adolescents don’t come in for well visits.” Clinician participants reported that they were often busy taking care of acute care issues, which limited the amount of time available to initiate discussion about vaccination. As one participant explained “Providers may be hesitant to recommend the vaccine because they have a very limited amount of time with patients and don’t want to get caught up in a 15-min conversation about one vaccine.” Further, participants identified barriers related to the lack of infrastructure and support for prompts to initiate provider recommendation when a patient was due for HPV vaccination. While most practices used an electronic health record (EHR), few to none had invested in developing electronic prompts for HPV vaccine dose reminders. Successful practices manually identified patients, which was cumbersome and labor intensive. The lack of prompts was attributed to limitations of EHR systems. One participant stated “Most IT EHR systems do not have population management systems in them. For some, it is an add-on that needs to be purchased, so not every practice has the capability.” Another participant further explained “For some centers, staff time is the limitation to create reminder and recall templates.” Reimbursement for the cost of administering the HPV vaccination was not a problem for most practices, but may have posed a barrier for some smaller practices. As one participant explained “Some vaccines you get reimbursed less than the actual vaccine cost. Larger practices have more power to negotiate these costs.” Another participant described “Administrative fees have come up. Some clinics have to carve out vaccines because they don’t get full cost back.” Recent pharmacy legislation in SC has resulted in pharmacists being allowed to vaccinate adolescents with prescription and adults without prescription. Participants noted that this could improve access to HPV vaccination for both target populations. However, a participant from the state pharmacy association explained “An issue in the pharmacy community is that not all insurance companies will reimburse pharmacists,” which posed a barrier for utilizing pharmacy-based vaccination.

### 3.2 Facilitating factors

The main identified facilitating factors specific to our state were 1) a high degree of momentum in commitment to addressing HPV vaccination uptake, 2) the school-entry diphtheria, tetanus, and acellular pertussis (Tdap) requirement, 3) pharmacist-administered HPV vaccination, 4) the SC state immunization registry and other IT infrastructure, 6) HEDIS and other reportable quality measures, and 7) the federal VFC program and state vaccine program as funding sources for HPV vaccination that could be further leveraged. Each of these is discussed in detail below ( Table 3).

**Table 3:** Facilitating factors for HPV vaccination reported by participants.

**Themes**	**Quotes**
**Momentum in commitment to addressing HPV vaccination uptake**	
National level	– President's Cancer Panel Report in 2014 – Patient Centered Medical Home standards require pediatric practices to conduct 6 QI projects and implement behavioral health.
State level	– There is leverage behind cervical cancer. With Cervical Cancer Free South Carolina, the work being done at the Medical University of South Carolina, the University of South Carolina, the South Carolina Cancer Alliance, many organizations are targeting cancer. These are wonderful resources not every state has. – Momentum is going for us, this grant, state health department money, health disparities money. We have a real opportunity to tip the scale. Is it sustainable? I think once you normalize the behavior, I didn’t turn green; it’ just the norm. Parents and kids don’t run screaming when they hear the term “HPV.” – We are working hard on the cervical cancer prevention act to add HPV vaccination to the state vaccine program. – One of our greatest strengths is our South Carolina American Academy of Pediatrics. They have the QTIP grant which “stands for Quality through Technology and Innovation in Pediatrics. SC was one of 10 states to receive a grant. The project includes about 18 practices in South Carolina, with 6 of their sites focusing on process improvement to increase HPV vaccination”
**School-entry Tdap requirement**	
Opportunity to introduce/promote all ACIP recommended vaccines	– The state health department mailed a flyer to parents to notify them of the Tdap requirement for middle school entry; it informed parents of other recommended vaccines such as the HPV vaccine. This was the 1st time some parents heard of HPV vaccine. – Using Tdap as the hook for HPV vaccination is the key. When children have to get the mandatory Tdap vaccine for entry to middle school, that is when you can direct them to their provider to get their HPV vaccinations. – Between 10 and 13 they have to get that Tdap for school; that is the best bet for the first one.
**Pharmacist-administered HPV vaccination**	
Increased access	– The fact that pharmacist may be able to provide any vaccine with a prescription is a plus from my standpoint… We have more immunizing providers than some other states.
**SC immunization registry**	
Registry is well-accepted by providers	– I love the fact that we have a registry. I think that's an excellent tool to be able to determine who is vaccinated and to prevent duplicate vaccination and to keep track of actual vaccination rates.
**HEDIS and other reportable measures**	
Linking reimbursement to NCQA HEDIS measures would create pressure to vaccinate	– HPV vaccination is a Healthcare Effectiveness Data and Information Set (HEDIS) 2015 measure. – 2014 National Committee on Quality Assurance (NCQA) standards put more emphasis on prevention; they must look at 2 vaccines at 2 different age groups. – HPV vaccination is a NCQA HEDIS measure. It's not one the DHHS has chosen as a withhold measure. – Providers like to get good grades and to get paid well. You could tie vaccination rates to reimbursement. Providers will increase HPV vaccination rates just because they are being rated.
**Existing funding for HPV vaccination**	
Federal VFC Program	– We only order about 2/3 of the doses of HPV vaccine available through the federal Vaccines for Children program.
State Health Department	– The state health department has the money to include coverage of the HPV vaccine through the State Immunization Program. – The state health department can cover the vaccination without legislation (but they don’t).

#### 3.2.1 Momentum in commitment to addressing HPV vaccination uptake

Enthusiasm about momentum at the national level was fueled by the President's Cancer Panel Report in 2014, the Cervical Cancer-Free America movement, and federal funding to address HPV vaccination efforts available through the NCI and CDC. One participant summarized this momentum saying “Momentum is going for us—this grant, state health department money, health disparities money. We have a real opportunity to tip the scale.” At a state level, Cervical Cancer Free SC, a state partner of the national advocacy organization committed to elimination of cervical cancer through vaccination, screening, and education, had made efforts to promote coordination among state players. As one participant explained “There is leverage behind cervical cancer. With Cervical Cancer Free South Carolina, the work being done at the Medical University of South Carolina, the University of South Carolina, the SC Cancer Alliance; many organizations are targeting cancer. These are wonderful resources that not every state has.” Clinician participants reported that some pediatric practices were participating in a statewide quality improvement project of the American Academy of Pediatrics to increase vaccination uptake. One participant described that SC was one of 10 states to receive a grant to focus on pediatric QI initiatives, including HPV vaccination. She explained “The project includes about 18 practices in South Carolina, with 6 of their sites focusing on process improvement to increase HPV vaccination.” Finally, at the time of the interviews, the state legislature was considering the Cervical Cancer Prevention Act, which would provide education about the HPV vaccine to parents of middle school children and add HPV vaccination to the state vaccine program. As one participant explained “We are working hard on the Cervical Cancer Prevention Act to add HPV vaccination to the state vaccine program.”

#### 3.2.2 School-entry TdaP requirement

Participants reported that the Tdap vaccination requirement in SC provided a “hook” to initiate HPV vaccination. The Tdap vaccine recently became an entry requirement for middle school in SC. The state health department sent a flyer to parents noting the new Tdap requirement and promoting all adolescent vaccines recommended by the ACIP, including the HPV vaccine. One participant described “This was the first time some parents had heard of the HPV vaccine.” The Tdap requirement also offered an opportunity for providers to introduce all ACIP recommended vaccines, which was especially critical for adolescents who do not participate in well-child visits. As one participant explained “Using Tdap as the hook for HPV vaccination is the key. When children have to get the mandatory Tdap vaccine for entry to middle school, that is when you can direct them to their provider to get their HPV vaccinations.”

#### 3.2.3 Pharmacist-administered HPV vaccination

An opportunity exists in SC for HPV vaccination in pharmacies. Pharmacies offer increased access points for completion of the three-dose HPV vaccination series and enable young adults to be vaccinated without visiting a physician. As one participant described “The fact that pharmacists may be able to provide any vaccine with a prescription is a plus from my standpoint. We have more immunizing providers than some other states.” Participants also noted the importance of integration across systems (i.e., coordination of vaccination records between pharmacies and the medical home). One participant explained “An important component is for the pharmacist to put the vaccination in the DHEC immunization registry to ensure continuity of care/recordkeeping.” One participant indicated that pharmacies have robust built-in mechanisms to transfer patient data back and forth to other entities, such as to patient medical homes.

#### 3.2.4 SC immunization registry

At the health system level, participants noted the opportunity to build on recent improvements in infrastructure to support HPV vaccination. The state vaccine registry provides a new clinical tool to coordinate vaccination across multiple settings, including pharmacies. Starting in January 2017, all providers have been required by law to enter to record vaccinations in the registry [37]. Interviews with clinician participants revealed most providers were satisfied with the immunization registry and regularly entered vaccinations administered into the registry. Participants noted the value of the registry as a surveillance tool to identify regions of the state and clinical settings with low vaccination rates that could be targeted for special interventions to support HPV vaccination. As one participant described “I love the fact that we have a registry. I think that's an excellent tool to be able to determine who is vaccinated and to prevent duplicate vaccination and to keep track of actual vaccination rates.” State agencies and private companies could be resources to help practices implement system recalls and reminders for HPV vaccinations, as they have helped to pay for installing these systems in the past. One participant recommended “Work with pharmaceutical companies to help fund reminder/recall systems.”

#### 3.2.5 Health effectiveness data and information set (HEDIS)

HPV vaccination was a reportable HEDIS measure for adolescent females, which participants described as a facilitating factor for vaccination. The planned inclusion of adolescent males for the HPV vaccination measure was noted as beneficial. The HEDIS measure for HPV vaccination was reportedly important for exerting pressure on practices to measure performance and meet certain standards. As one participant explained “The 2014 National Commission for Quality Assurance (NCQA) standards put more emphasis on prevention. They (providers) must look at 2 vaccines at 2 different age groups.” Further, HEDIS measures could encourage practices to invest their limited time and energy in improving HPV vaccination rates. Participants similarly mentioned that linking reimbursement to HEDIS measures could create even more positive pressure for improvement. One participant described “Providers like to get good grades and to get paid well. You could tie vaccination rates to reimbursement. Providers will increase HPV vaccination rates just because they are being rated.”

#### 3.2.6 Existing funding sources for HPV vaccination

Participants noted that the federal VFC program in SC has the opportunity to access more funds for HPV vaccines, which could be a facilitating factor. One participant explained that the number of vaccine doses available to each state is based on the number of individuals within the recommended age group for vaccination, and that “SC uses only about 2/3 of allotted doses of HPV vaccines available through the federal VFC program.” Thus, free HPV vaccine doses are available to vaccinate more children who are eligible for the VFC program (i.e. those who are uninsured or who are on Medicaid). Further, participants reported that the state health department could include HPV vaccines in the state immunization program without additional legislation and that they have funds available to do so. If this policy change were made, it would enable coverage for “underinsured” children, or those who are ineligible for Medicaid, but who do not have health insurance.

### 3.3 Strategies to increase HPV vaccination in South Carolina

As shown in Table 4, participants described several strategies to increase HPV vaccination in SC. Many of these strategies build on facilitating factors, while others were grounded in activities undertaken in other states or in other fields, such as tobacco control, where relevant best practices may be transferable. Promising strategies for improving HPV vaccination fell into three general categories: 1) addressing lack of awareness about the importance of HPV vaccination among general public and providers; 2) advocating for policy changes around coverage of HPV vaccines, vaccine education, and pharmacy-based vaccination; and 3) robust coordination of state stakeholder efforts.

**Table 4:** Strategies for HPV vaccination reported by participants.

**Themes**	**Quotes**
**Addressing lack of awareness among general public and providers**	
Widespread public education about HPV vaccination via local engagement, mass media and/or social media	– Develop a public service program to try to educate the population that this is a cancer prevention vaccine. – There needs to be a statewide education program explaining why this vaccine is necessary and that its safe/effective. – Direct to parent flyer about adolescent vaccines really helped to introduce the concept in the state. – Address HPV vaccination barriers linked to concerns about children's sexual activity through comprehensive, age-appropriate health education. – Peer to peer pressure is key. We rolled out the Rage against the Haze campaign across the state. We used teens as peer educators in schools. You give kids something and they run with it. Some of these kids are incredibly intelligent. They can take any message and figure out how to make other people listen. Plant some seeds with the kids who plant some seeds and then you have a network of youth. Could do the same with HPV. – Mobile texting campaigns are underused and can have a real benefit. For example, Text for Baby is a phenomenal campaign that has had great outcomes.
Targeted education of healthcare providers through in-service sessions at state provider meetings, email listservs, and CME credit programs	– Training for providers would be key. – Pediatricians have to be on board to send the right message. – Comprehensive educational marketing that could be like a toolkit of resources could be given to school nurses, pediatricians and family clinics to make it easier to educate parents. We need a way to catch people's attention and have the information come from somebody they trust like their healthcare providers and school nurses. – Giving physicians a protocol to get the entire staff organized and working together for HPV vaccination would be great, especially on how to phrase things compared with the CDC form.
Synergistic effect of targeting these two groups	– It's two-pronged: part of it is the clinicians themselves and how they offer or inform the family that their child needs a vaccine; the other prong is the parent asking for it. – Education is key to getting vaccination rates up. It needs to be across the board and include providers and parents. – Doctors and the media need to strongly encourage the vaccine. – The doctor promoting it is key; then how do you get the message to parents for them to know this is out there?
**Advocating for policy changes around HPV vaccination**	
Advocating for policy to expand pharmacy HPV vaccination	– Make every provider a vaccinator, such as pharmacists. – If pharmacists could give the vaccine, we could reach some of those nooks and crannies in the state that are underserved by providers. We have shown a bump in flu and other vaccines since pharmacists started vaccinating. – We (state pharmacists) are trying to get listed as a provider for the Vaccines for Children program.
Policy advocacy	– Use the South Carolina Cancer Alliance for lobbying and for the advocacy piece. – We need a public health policy to require the state health department to include the HPV vaccine in the state vaccine program. – There needs to be a steady lobby for legislation to work. Look into other policy options instead of legislation. – Get community buy-in. We need religious leaders who understand the science and have connections to organizations like the SC Policy Council, SC Palmetto Council, SC Catholic Coalition, SC Baptist Convention. That speaks volumes legislatively and in churches back home. – The more people understand the HPV vaccine, the more they will advocate for legislation.
**Coordination of efforts among SC stakeholders**	
Public education/engagement	– Work with Parent Teacher Associations (PTSs); Engage the faith community. If they approve things in the state, people may be more likely to get vaccinated. Show the community that it's not something evil. – The Witness Project (grassroots state cancer screening initiative) can really help in the AA community, which is a matriarchal society. We have to convince grandmom before mom and daughter. Person to person is how you get information out across SC. – Churches are a big deal around here; take it into the black communities. They promote all types of health stuff in the churches. If you could educate them, they could educate their parishioners that this is a cancer vaccine. – Using the school system. Getting school nurses to provide information and answer parents’ questions. Parents and students already have a relationship with those people. – We have an active peer education group on campus. It has worked very well. Students listen to their peers. – One thing that has worked with tobacco control is local coalitions. We should get some strong women to come out and support HPV vaccination in their communities. – Get pharmaceutical companies to help spread the message: it is what they do best.
Provider education	– Provider organizations have been a force. Put pressure on providers to work on HPV quality measures. – CME's through the South Carolina Medical Association would be an opportunity to encourage HPV education at meetings. – We at the SC American Academy of Family Practitioners would get involved with an intervention (parents giving the gift of life) and getting this information to our physicians. There would be opportunity to do something with HPV and CME at the meeting. – It may help to go through organizations like the American Academy of Pediatrics who endorse the vaccine. – At one of our Federally Qualified Health Centers (FQHC) conferences, you could do sessions on HPV vaccination. – If you have crafted something easy and simple, you can make it a webinar offering CME credits. – Work with pharmaceutical companies to help with both reminder and recall systems and provider education.
Health system prompts	– Our system requires providers to notify front desk staff to schedule future appointments for the second and third doses. Recall systems just become a part of daily practice. – Work with pharma companies to help with both reminder/recall systems and provider education. – Meaningful use pays health centers to create IT infrastructure for preventive care such as vaccination. This could be used to encourage immunization recall/reminder systems.
Coordination and tracking	– I think it would be important to invest in Cervical Cancer Free South Carolina as a home for coordinating these efforts. – HPV could be promoted through South Carolina Cancer Alliance (SCCA). It could be the topic for one of the 3 SCCA meetings held each year. – SC does not suffer a dearth of resources. It suffers a dearth of collaboration of those resources. I think that represents the best opportunity for improvement for the foreseeable future for anything, and especially for HPV vaccination.

#### 3.3.1 Addressing lack of awareness among general public and providers

To address the lack of awareness of HPV vaccination among the general public and providers, a two-pronged educational strategy was recommended: 1) widespread public education about HPV vaccination via mass media and/or social media and 2) targeted education of healthcare providers through in-service sessions at state provider organization meetings, email listservs, and CME credit programs. Many participants noted that together parents and providers represent the driving force for HPV vaccination, and that engaging both these target groups simultaneously could be synergistic in facilitating readiness for HPV vaccination during the adolescent healthcare visit. As explained by one participant “It's two-pronged: part of it is the clinicians themselves and how they offer or inform the family that their child needs a vaccine; the other prong is the parent asking for it. Another participant noted ”Education is key to getting vaccination rates up. It needs to be across the board and include providers and parents.”

#### 3.3.2 Advocating for policy changes around HPV vaccination

From a policy standpoint, it was recommended that organizations work together to support: 1) the Cervical Cancer Prevention Act, which was designed to enable education of middle school parents and coverage of HPV vaccines for underinsured adolescents, 2) policy change in partnership with insurers to cover the HPV vaccine among young adults in the catch-up vaccination group, and 3) legislation expanding pharmacy legislation, such as by allowing pharmacists to vaccinate adolescents without a prescription. Multiple participants cited the success in increasing flu vaccination through pharmacy-based administration. As one participant described “If pharmacists could give the vaccine (without a physician prescription for adolescents), we could reach some of those nooks and crannies in the state that are underserved by providers. We have shown a bump in flu and other vaccines since pharmacists stated vaccinating.” One stakeholder stressed that advocating for more of the major state insurers to cover pharmacy-based vaccination was a crucial step in utilizing that resource. She explained “We (state pharmacists) are trying to get listed as a provider for the VFC program.”

#### 3.3.3 Coordination of efforts among SC stakeholders

Purposeful coordination of efforts between state partners was also recommended as a strategy to improve HPV vaccination rates. One participant suggested “I think it would be important to invest in Cervical Cancer Free South Carolina” to coordinate these efforts. Another participant suggested “It may help to go through organizations like the American Academy of Pediatrics who endorse the vaccine.” This coordination would enable the concurrent implementation of statewide public and provider education, coupled with grassroots advocacy work to garner support for the HPV vaccine at the local community level. Universities, public health organizations, and provider organizations were noted to have the skillset needed to conduct high quality public and provider education. One participant explained “We could use assistance with wording and support for vaccination from our state partners such as the Hollings Cancer Center. We are the providers, but it could be a statewide initiative supported strongly by state partners.” Partnerships with local schools, churches, and other community organizations were recommended to reinforce the message at the local level that HPV vaccination is a safe and effective method for preventing cancer. Participants made suggestions such as “Use the school system. Get school nurses to provide information and answer parents’ questions. Parents and students already have a relationship” and “Work with Parent Teacher Associations; engage the faith community. If they approve things in the state, people may be more likely to get vaccinated.” Another participant described “Get community buy-in. We need religious leaders who understand the science and have connections to organizations like the SC Policy Council, the SC Palmetto Council, the SC Catholic Coalition and the SC Baptist Convention. That speaks volumes legislatively and in churches back home.” To improve vaccination rates at the practice level, participants advised working with organizations such as pharmaceutical companies and the South Carolina Office of Rural Health that can provide funding and/or technical support for creation of health system prompts. One participant recommended “You can work with pharma companies to help with both reminder/recalls systems and provider education. They want their vaccine out there. Pharma can help link EHR to generate reminder/recalls.” One participant described that the SC Office of Rural Health has “done a lot of work in our state to get our providers using electronic medical records. I wouldn't say its 100% but the vast majority now are using electronic health records and are attesting to meaningful use.” A few participants suggested that state agencies or provider organizations provide practices with a standard toolkit to further facilitate HPV vaccination. As one participant explained “Comprehensive educational marketing that could be like a toolkit of resources could be given to school nurses, pediatricians and family clinics to make it easier to educate parents. We need a way to catch people's attention and have the information come from somebody they trust like their healthcare providers and school nurses.” On the policy level, coordinated advocacy would help achieve needed changes. One participant recommended “Use the South Carolina Cancer Alliance for lobbying and for the advocacy piece.” Another participant explained “One thing that has worked with tobacco control is local coalitions. We should get some strong women to come out and support HPV vaccination in their communities.” Another participant added additional insight, saying “The more people understand the HPV vaccine, the more they will advocate for legislation.”

## 4 Discussion

A statewide environmental scan was carried out to assess barriers, facilitators and strategies for improving HPV vaccination that are contextually specific to SC. As anticipated, the environmental scan identified many of the same types of barriers that have been previously identified nationally. For example, barriers were identified at the patient (e.g., lack of vaccine awareness, concerns about vaccinating adolescents for an STD, lack of physician recommendation), provider (e.g., lack of time to discuss vaccine, infrequency of doctor visits for adolescents, lack of vaccine reminder/recall systems), and policy (e.g., HPV vaccine is not mandatory for school entry or provided in schools) levels.

More importantly, stakeholder interviews also revealed actionable information about these barriers that were contextually specific to SC. For example, lack of investment in state-level public/provider awareness campaigns was commonly identified as a problem. While vaccine cost was not identified as a common barrier among younger adolescents, lack of vaccine coverage was a barrier for “underinsured” adolescents and young adults because the state vaccine program and some insurers, respectively, did not cover vaccination for these populations at the time of this project. In addition, while pharmacists in the state could vaccinate adults without prescription and adolescents with prescription, lack of coverage for pharmacy-based vaccination by insurers and the requirement for physician prescription in order for pharmacists to vaccinate adolescents limited the potential impact of pharmacy-based vaccination. Finally, politicization of HPV vaccination related to concerns about sexuality and promiscuity had stifled efforts to make the vaccine mandatory or provide school-based vaccination or education about the vaccine. Thus, the stakeholder interviews provided a rich source of information about specific and actionable barriers to HPV vaccination.

To address these barriers, the environmental scan identified facilitators that can be leveraged to improve HPV vaccination rates in SC, including demonstrated commitment from state partners, the school-entry Tdap requirement, pharmacy-based HPV vaccination, EHR infrastructure in many practices that could support reminder/recall systems, and untapped funding sources for HPV vaccination. Suggestions about strategies for improving HPV vaccination included multi-level educational efforts that combine state level awareness campaigns and engagement with grassroots organizations such as churches and schools, policy advocacy, and purposeful coordination of efforts among state stakeholders to methodically address each of the barriers to HPV vaccination.

To enable comparison of our results with those from other NCI-funded HPV environmental scan projects, we identified five studies that reported environmental scan results. These studies were conducted in diverse parts of the US, including one western US region that included 10 states [38], Utah [39], Los Angeles, California [40], Texas [41], and Tennessee [42], representing diverse populations in terms of rurality/urbanity, level of religiosity and race and ethnicity. These studies were conducted via qualitative interviews or focus groups, cross-sectional surveys or a combination of these methods. Interestingly, three findings were pervasive across our study and these comparison studies. Four of the five studies explicitly reported lack of parental knowledge about the HPV vaccine and lack of provider recommendation as critical barriers to HPV vaccination [38], [39], [40], [41], and all five of the studies reported concerns about vaccination of adolescents for a sexually transmitted disease as a barrier to HPV vaccination [38], [39], [40], [41], [42]. Interestingly, we anticipated that concerns about sexuality and promiscuity might be more common in southern states such as SC, but these concerns were identified in all five of these studies that reported on HPV environmental scan project results that were conducted in diverse parts of the country. Similar to our results, common strategies recommended for improving HPV vaccination focused on the need for policy change (e.g. mandatory vaccination, school-based vaccination) [38], [39], [40], [42], refining the HPV vaccination message to overcome critical knowledge gaps (e.g. HPV vaccine disease rates, vaccine as cancer prevention, the need to vaccinate boys) [38], [39], [40], [41], [42], using local coalitions as infrastructure for networking, sharing ideas and voicing concerns related to vaccination [38], [40], [42] and gaining buy-in from religious leaders to overcome concerns about promiscuity [38], [42]

In contrast to our study, the three environmental scans conducted in settings that served a high volume of medically underserved or multi-cultural/lingual patients identified additional needs for reading-level, language, and culturally-tailored health education materials [40], [41], [42]. Two of these same studies also identified challenges in verbally communicating about HPV vaccination with clients who spoke another language [40] and the need for more onsite interpreters [42], given the complexity of HPV vaccination communication.

NCI's strategic decision to fund environmental scans across the US demonstrates a strong commitment to ensuring that critical cancer prevention methods such as HPV vaccination move beyond research into communities across the country. These projects provided many NCI-designated and comprehensive cancer centers with dedicated resources to help understand and develop contextually appropriate strategies for optimizing HPV vaccination for cancer prevention. Cancer centers and health science universities are uniquely poised to facilitate coordinated statewide efforts to increase HPV vaccination for many reasons. First, these organizations often have the respect among those in their catchment areas as being their state or region's experts in residence on HPV vaccination. This was a common comment by participants in the current study. Second, these organizations may have a diverse cadre of researchers and clinicians with relevant expertise and interest to facilitate statewide efforts to increase HPV vaccination. For example, cancer centers and health science universities have professionals working in epidemiology and biological aspects of HPV vaccination, health education and behavioral sciences, dissemination and implementation, community engagement, health systems and policy and program evaluation. In addition, oncologists who care for patients with HPV-related cancers are often passionate to find ways to prevent these cancers in the future. Third, there are potential federal funding mechanisms, such as the environmental scan grant that funded the current project, that can be utilized to help support the work of NCI-affiliated cancer centers to facilitate HPV vaccination coordination efforts. Many opportunities exist for cancer centers and health science universities to help facilitate HPV vaccination through organizations such as state cancer alliances and non-profit organizations such as Cervical Cancer Free America.

Since the time of this environmental scan, several accomplishments have occurred in SC related to the barriers and strategies identified by participants. The Cervical Cancer Prevention Act, which was stalled at the time of interviews, was passed in SC in April 2016 [43]. The policy states that all middle schools are required to educate parents about HPV vaccination and that underinsured adolescents can receive HPV vaccination through the State Vaccination Program. Momentum in commitment to HPV vaccination at the state level was demonstrated by a press release from the Hollings Cancer Center, SC's sole NCI designated cancer center, which encouraged HPV vaccination and was signed by over 25 state partners. Finally, ACIP and the CDC approved a 2-dose schedule for HPV vaccination for adolescents under the age of 14 in October 2016 [44]. This not only alleviates barriers to completing the 3-dose series, but also encourages physicians to recommend vaccination earlier because the 2-dose series is only approved for adolescents who begin the vaccination series between the ages of 9 and 14.

For further progress on HPV vaccination, sustained and coordinated efforts are required in SC. This environmental scan effectively evaluated the current barriers and facilitators to timely HPV vaccination in SC. This project also generated information that will be helpful in designing contextually appropriate strategies for improving HPV vaccination across the Southeast, where HPV vaccination rates are lower than many other regions of the US [1].

## Disclosure of funding

This project was funded by the National Cancer Institute through grant #3P30CA138313-06S2.

## Footnotes

Appendix A Supplementary data associated with this article can be found in the online version at doi:10.1016/j.pvr.2017.11.003.
