# Supplementary material for: Barriers, facilitators, and potential strategies for increasing HPV vaccination: A statewide assessment to inform action
Source: Papillomavirus Res. 2017 Dec 7;5:21–31. doi: 10.1016/j.pvr.2017.11.003 (PMC5886972; doi:10.1016/j.pvr.2017.11.003)
Supplement: Supplementary file 1 — Supplementary material [file mmc1.pdf]

Appendix: HPV Vaccination Key Stakeholder Interview Script

A. Introductions: *Hi, my name is* ___________________ *and I work with the (Center Name). We are carrying out a project to find out more about what is influencing human papillomavirus (or HPV) vaccination in our state. We also want to learn about strategies that could help to improve HPV vaccination rates in our state. We really appreciate your taking the time today to share your thoughts about HPV vaccination.*

1. To begin, could you please tell me a little bit about your work?

B. Perceptions about Barriers and Facilitators to HPV Vaccination: *Next, I'd like to ask a few general questions about your experience with HPV vaccination.*

1. What are your thoughts about HPV vaccination in our state? (potential probe: what role do you think the HPV vaccine will play in reducing new cases of cervical cancer in our state?)

2. What factors in our state facilitate HPV vaccination? (Probe for and discuss up to 3 facilitators; List each facilitator)

   *Now thinking about the **1st facilitator** you mentioned, how can this facilitator be expanded to enhance statewide HPV vaccination rates? (Repeat for each facilitator)*

3. What are the barriers to HPV vaccination in our state? (Probe for and discuss up to 3 barriers; List each facilitator)

   *Now thinking about the **1st barrier** you mentioned, how can this barrier be addressed in our state? (Repeat for each barrier)*

C. Recommendations for HPV Vaccination Strategies: *A goal of our project is to learn about strategies that may help to improve HPV vaccination rates in our state. We want to learn from any experiences that you have had with either general or HPV vaccine specific strategies that might work to improve HPV vaccination rates*

1. What strategies are you aware of that have been used to improve vaccination rates in general? (Probe for and discuss up to 3 strategies; List each strategy)

   *Now thinking about the **first strategy** you mentioned: Can you tell me more about this strategy?*

   How well did this strategy work; How could this strategy be improved? (Repeat for each strategy)

2. What type of strategy or strategies do you think would be most effective specifically for improving HPV vaccination rates in our state? (Probe for and discuss 3 strategies; List each strategy)

   *Now thinking about the **first strategy** you mentioned: Can you tell me more about this strategy? (Repeat for each strategy)*

3. Policy/legislation as a means to increase HPV vaccination can range from no proactive policies to awareness campaigns and all the way to mandating vaccination for school attendance.

   What are your preferences about the level of policy/legislation to increase HPV vaccination in our state?

   Thinking about policy/legislation, what specific strategy would you suggest to increase vaccination rates?

D. HPV Vaccination Linkages with Statewide Stakeholders: *An important goal of our project is to establish statewide linkages that can be used to improve HPV vaccination rates in our state.*

1. Tell me about any collaboration that you've been involved with to improve HPV vaccination in the state, and briefly assess the effectiveness of the collaboration in accomplishing this goal.

2. Who do you think are the key stakeholders who should be included in developing strategies to maximize HPV vaccination rates? (Probe for up to 3 stakeholder groups; List each stakeholder group)

   *For the **first stakeholder group** you mentioned, what discussions are needed to move HPV vaccination forward in the state? (Repeat for each stakeholder group)*

E. Add Stakeholder Specific Module(s) as Appropriate:

E1. Provider Organizations. *Next, I'd like to ask your thoughts about HPV vaccination practices among providers in your professional organization.*

1. *Providers are sometimes uncertain about whether to offer the HPV vaccine to patients.* What are reasons that you or other providers may be hesitant to offer HPV vaccination to patients?

   For what groups of patients does this barrier apply? (probe for specific groups of patients)

   What do you hear most often from patients and their family members about HPV vaccination?

2. *Nationally, some providers report cost and lack of insurance coverage pose a barrier to vaccinating children against HPV.* What can you tell me about any cost or reimbursement issues that you have heard providers in our state discuss?

   What is the cost issue? (probe for reimbursement issues)

   For which groups or patients do you find cost to be a problem?

   Do you have any ideas about how this issue could be fixed?

3. *As you know, patients often receive care from multiple providers. In our state in 2014, an immunization registry was set up so that providers across multiple practices can record vaccines given.* What are your thoughts about having this immunization registry in our state?

4. *The FDA has now approved the Gardasil 9 vaccine. It protects against the HPV virus strains that cause up to 90% of cervical cancer.* How do you think this will impact HPV vaccination rates?

5. What else about your provider organization and HPV vaccination is important for us to know?

E2. Insurers. *Next, I'd like to ask you a few questions about HPV vaccination in your insurance company.*

1. Can you describe your organization's policy for HPV vaccination?

2. To what extent is improving HPV vaccination rates a priority for your organization?

3. Can you tell me about any QI strategies that you have in place to improve performance?

   For the **1st strategy** you mentioned, what are your thoughts about the effectiveness of this strategy? (Repeat for each strategy)

4. Can you tell me about any QI strategies that you have in place to improve HPV vaccination performance?

    What are your thoughts about the effectiveness of this strategy?

5. What else about insurance coverage and HPV vaccination is important for us to know?

E3. Public Health Immunization Program. *Next, I'd like to ask you a few questions about public health programs that can support HPV vaccination.*

1. What impact do you feel that the statewide immunization registry will have on vaccination in our state?

   What about the adolescent vaccinations/HPV vaccine specifically?

2. How do you feel that the state vaccination program influences vaccination in our state?

   How do you feel the state vaccination program impacts adolescent immunization?

4. What else about the public health immunization program and HPV vaccination is important for us to know?

E4. School System. *Next, we'd like to ask about how HPV vaccination could be encouraged in our schools.*

1. What has been your experience with HPV vaccination of adolescents in the state school system, if any?

2. Can you tell me about any HPV education that is included as part of the state school health curriculum?

3. How do you feel that schools could increase rates of recommended adolescent vaccines?

4. Can you tell me about any vaccine clinics where students can be vaccinated at school?

   If so, how did these clinics worked? Prompts: opt-in vs. opt-out?

   Did you work with public health on these vaccine clinics?

   Do you feel school-based vaccination clinics would increase rates of adolescent vaccination?

5. What else about the school system and HPV vaccination is important for us to know?

E5. Legislation: *Next, we would like to ask about HPV vaccine legislation in our state.*

1. What are your thoughts about previous proposed HPV vaccination legislation in our state that was not passed?

   What do you think was good about this legislation?

   What were your biggest concerns about the legislation?

   Was there anything that could have been changed in the legislation so that it could have been passed?

2. What type of HPV-vaccine related legislation do you think is needed?

   What types of legislation do you think our state legislators would support?

3. *HPV vaccination legislation will be introduced in both the Senate and House this year. Review the bill. Address any questions regarding specifics of the bill.*

   Do you think this is legislation that your constituents would support? Why or why not?

4. The HPV vaccination is the only ACIP-recommended adolescent vaccine that is not included in the state vaccine program. Would you (or your constituents) support legislation to include this in the state vaccine program?

5. What else about HPV legislation and policy is important for us to know?

Final Question:

1. What else about HPV vaccination is important for us to know?

This is all the questions we have for you today. Do you have any questions before we end? Thank you so much for your willingness to spend some time talking with us today!
